# Substitution of low-risk skin cancer hospital care towards primary care: A qualitative study on views of general practitioners and dermatologists

**DOI:** 10.1371/journal.pone.0213595

**Published:** 2019-03-19

**Authors:** E. C. Noels, M. Wakkee, R. R. van den Bos, P. J. E. Bindels, T. Nijsten, M. Lugtenberg

**Affiliations:** 1 Department of Dermatology, Erasmus MC Cancer Institute, Rotterdam, the Netherlands; 2 Department of Public Health, Erasmus University Medical Center, Rotterdam, the Netherlands; 3 Department of General Practice, Erasmus University Medical Center, Rotterdam, The Netherlands; University of Birmingham, UNITED KINGDOM

## Abstract

**Background:**

Rising healthcare expenditures places the potential for substitution of hospital care towards primary care high on the political agenda. As low-risk basal cell carcinoma (BCC) care is one of the potential targets for substitution of hospital care towards primary care the objective of this study is to gain insight in the views of healthcare professionals regarding substitution of skin cancer care, and to identify perceived barriers and potential strategies to facilitate substitution.

**Methods:**

A qualitative study was conducted consisting of 40 interviews with dermatologists and GPs and three focus groups with 18 selected GPs with noted willingness regarding substitution of skin cancer care. The interviews and focus groups focused on general views, perceived barriers and potential strategies to facilitate substitution of skin cancer care, using predefined topic lists. All sessions were audio-taped, transcribed verbatim and analyzed using the program AtlasTi.

**Results:**

GPs were generally positive regarding substitution of skin care whereas dermatologists expressed more concerns. Lack of trust in GPs to adequately perform skin cancer care and a preference of patients for dermatologists are reported as barriers by dermatologists. The main barriers reported by GPs were a lack of confidence in own skills to perform skin cancer care, a lack of trust from both patients and dermatologists and limited time and financial compensation. Facilitating strategies suggested by both groups mainly focused on improving GPs’ education and improving the collaboration between primary and secondary care. GPs additionally suggested efforts from dermatologists to increase their own and patients’ trust in GPs, and time and financial compensation. The selected group of GPs suggested practical solutions to facilitate substitution focusing on changes in organizational structure including horizontal referring, outreach models and practice size reduction.

**Conclusions:**

GPs and, to lesser extent, dermatologists are positive regarding substitution of low-risk BCC care, though report substantial barriers that need to be addressed before substitution can be further implemented. Aside from essential strategies such as improving GPs’ skin cancer education and time and financial compensation, rearranging the organizational structure in primary care and between primary and secondary care may facilitate effective and safe substitution of low-risk BCC care.

## Introduction

Rising health care expenditures places the potential for substitution of hospital care towards primary care high on the political agenda.[1] With the Dutch health care system being one of the most costly in Europe[2], the ultimate goal for the Dutch health care system to make it more sustainable for the future.[3, 4] This may imply substituting tasks to primary care, and preventing unnecessary referrals to secondary care. This potential solution is being explored by various countries, such as the UK and Australia.[5–9]

Implementation of change in practice, such as substitution of care, is a complex and iterative process; a large number of factors can hinder or facilitate the intended change. [10–13] An analysis of barriers to change among stakeholders is considered to be an essential first step. These barriers can subsequently be used to develop tailored strategies for implementation.[12] As acceptance by stakeholders is crucial for successful implementation, integrating the stakeholders’ preferences for strategies in the implementation plan may also be useful.[14, 15] After all, strategies to dispatch certain barriers need to be considered as appropriate by the stakeholders, as they are the ones executing the process.

Several areas have been defined as potential targets for substitution of care, among which is low risk skin cancer care.[4] Basal cell carcinoma (BCC) is the most frequent cause of skin cancer with an annual incidence rate of 37,637 in 2014.[16] Whereas high risk BCCs are considered appropriate for hospital care, low risk BCC may be suitable for primary health care. The recently issued guideline ‘suspicious cutaneous lesions’ of the Dutch College of General Practitioners supports this concept by providing recommendations for GPs on the diagnosis and treatment of low-risk BCCs.[17]

Currently, the stakeholders’ views concerning the potential of substituting low risk skin cancer hospital care to primary care are lacking. The aim of this qualitative study is to gain insight in healthcare providers’ views regarding substitution of skin cancer care. This includes an analysis of views on substitution, perceived barriers and an exploration of potential strategies. In addition, a subgroup of GPs with a noted willingness in skin cancer care is studied to identify practical solutions to facilitate substitution.

## Methods

### Study design

A qualitative study consisting of 40 semi-structured interviews with healthcare providers (18 dermatologists and 22 GPs), and three focus group meetings with a total of 18 selected GPs with noted willingness regarding substitution of skin cancer care was conducted. Individual interviews were considered the most appropriate for identifying views on substitution of skin cancer care, as they are suitable for identifying the physicians’ underlying motives and needs, and encourage participants to propagate their views and opinions freely.[18–20]

Focus group meetings were chosen to identify practical solutions to facilitate substitution of care as suggested by the selected GPs. By facilitating interaction between participants, focus groups enabled us to receive in-depth information and provide information on cognitions and motivations for strategies to dispatch barriers regarding substitution.[21]

### Selection of participants

#### Interviews skin cancer care providers

To select GPs and dermatologists for the interviews, purposeful sampling was used to obtain a knowledgeable group regarding skin cancer care, with willingness to communicate experiences and opinions.[22–25] Potential participants were selected from the authors’ personal network. In selecting participants we aimed to strive for variation in terms of years of professional experience, type of practice and hospital and level of urbanization. Initially, 5 GPs and 5 dermatologists were invited to participate in a pilot. This was ultimately extended to 22 GPs and 18 dermatologists in total. Invitations were sent by email, including an information leaflet about the study. Physicians could register for participation by contacting one of the researchers.

#### Focus groups selected GPs

To select GPs with noted willingness regarding substitution of skin cancer care for the focus groups, all GPs participating in the SKINCATCH trial were invited to participate by email. No overlap existed between this group of GPs and the GPs participating in the interviews. The SKINCATCH trial is a randomized controlled multicenter trial launched in 2016, which aims to evaluate the process of substitution of skin cancer hospital care towards primary care (Appendix A in S1 File). Participation in the trial was voluntary and the GPs participating in the trial have shown great enthusiasm and interest regarding substitution of skin cancer care from hospital care towards primary care.

All GPs participating in the trial received an information leaflet about the study and could register for participation in one of the three organized focus group meetings by contacting one of the researchers.

### Data collection

#### Interviews skin cancer care providers

The timing of the interviews was just prior to the issuing of the first Dutch primary care guideline on ‘suspicious cutaneous lesions’ (June 2017). All individual semi-structured interviews were conducted by an experienced independent qualitative researcher (ML) either in a face-to-face setting at a location of their preference (n = 28) or alternatively by telephone (n = 12). An interview guide, based on prior experience of the authors[14, 26], was used during the interviews, which included the following main themes: views on substitution of BCC care, perceived barriers, and potential strategies regarding substitution (Appendix B in S1 File). In addition to the topic of substitution of care, the interviews focused on the management of actinic keratosis, and basal and squamous cell carcinomas; this falls outside the scope of this paper. After the pilot, small changes were made to the topic guide although this did not concern the themes on substitution.

#### Focus groups selected GPs

The three focus group meetings were conducted between December 2017 and March 2018 at the Erasmus Medical Centre in Rotterdam. The sessions were moderated by one experienced independent qualitative researchers (ML) and an assistant, both not being involved in the SKINCATCH trial. One of the SKINCATCH trial researchers (EN) was present during the focus group meetings, but only to answer substantive questions regarding the trial.

In each focus group, a semi-structured discussion was held, subdivided in 2 parts: the first part focused on general views on substitution of care with a special emphasis on practical solutions to facilitate substitution of care. The second part of the focus groups focused on experiences with the SKINCATCH trial. The current study focusses on the first part (Appendix C in S1 File).

All interviews and focus groups were audio-recorded with consent of participants, and subsequently transcribed verbatim anonymously. The transcripts were imported to ATLAS.ti for analysis (version 8 Windows).

### Data processing and analysis

#### Interviews skin cancer care providers

An inductive approach to data analysis was applied drawing on elements of grounded theory (e.g., open coding, constant comparison).[21] Two researchers (ML and Mirjam Droger) independently coded the first 10 transcripts as part of the pilot. The obtained codes were discussed and adjusted, resulting in a preliminary code scheme. The remaining 30 transcripts were coded by one of the researchers (ML) using this coding scheme as a basis. New codes were added until needed. All coded transcripts were checked by a third researcher (EN) and differences were discussed and refined until agreement was reached. After 18 interviews with dermatologists and 22 interviews with GPs, thematic saturation, which was defined as little or no changes made to the codes, was reached in all covered research areas.[21]

The initial coding phase was followed by the phase of iterative and interpretive constant comparison. To identify emerging themes in terms of views, barriers and strategies, different codes were compared and clustered and the relationship between groups of codes was explored. This was done separately and independently for codes of GPs and dermatologists.

Inter-observer reliability was tested on several occasions, through coding of the first ten transcripts by more than one coder and by group discussions (on meaning) of codes and relationships between codes.

#### Focus groups selected GPs

Similar to the processing of the interview transcripts, two researchers (EN, ML) independently coded the first transcript after which the obtained codes were discussed and adjusted when needed. The resulting preliminary thematic coding frame was used for coding of all transcripts by one researcher (EN or ML) and checked by a second researcher (EN or ML). New codes were created when needed, and differences were discussed within the research team and refined until agreement was reached. Thematic saturation was reached after 3 focus group meetings. Subsequently, by iterative and interpretive constant comparison emerging themes were detected as the relationships between codes were explored.

### Ethical considerations

Ethical approval for the interviews and focus groups was granted by the medical ethics committee of the Erasmus University Medical Centre in Rotterdam (Interview study MEC-2016-204 and Focus group study MEC-2015-492). All participants have provided written informed consent.

This qualitative study has been designed and reported in accordance with the recommendations of the SRQR (Standards for Reporting Qualitative Research)(see S2 File).[27] This checklist provides a tool for transparent and comprehensive reporting of qualitative studies.

## Results

### Characteristics of participants

The participating sample of dermatologists and GPs had a representation from across the Netherlands. The dermatologists participating in the interviews consisted of 10 males and 8 females (Table 1). The median age was 45 years (interquartile range (IQR) 38–54). The majority worked in a peripheral hospital (n = 9). Of the 22 GPs participating in the interviews, 15 were males. The median age was 41 (IQR 38–53). Two physicians in the GP group were residents. The GP focus group size ranged from 4–8 participants with 41% males and a median age of 49 years (IQR 38–57).

**Table 1:** Characteristics of participants.

		Interviews		Focus groups
Characteristics		Dermatologists	GPs	Selected GPs
Total, n		18	22	17
Male, n(%)		10 (56)	15 (68)	7 (41)
Age, median (IQR)		45 (38–54)	41 (38–53)	49 (38–57)
Years of professional experience, median (IQR)		12 (6–20)	8 (6–23)	14 (8–25)
Setting, n(%)				
Dermatologist	Academic hospital	2 (11)	N/A	N/A
	Peripheral hospital	9 (50)	N/A	N/A
	ISTC	3 (17)	N/A	N/A
	Combination of the above	4 (22)	N/A	N/A
GPs	Individual practice	N/A	6 (27)	3 (18)
	Group practice	N/A	15 (68)	13 (76)
	Medical centre	N/A	1 (5)	1 (6)
Urbanisation degree, n(%)				
High-density		11 (61)	10 (46)	5 (29)
Middle high-density		5 (28)	5 (23)	6 (35)
Mediate density		2 (11)	1 (5)	6 (35)
Low density		0 (0)	3 (14)	0 (0)
Non-urban		0 (0)	3 (14)	0 (0)

Abbreviations: GP, general practitioner; IQR, interquartile range; ISTC, independent sector treatment center.

N/A: not applicable

### Views of health care providers regarding substitution of skin cancer care

#### Views of dermatologists

Aside from their general views, two main barriers and four potential strategies regarding substitution of care were identified for dermatologists (Table 2). Additional quotations illustrating each (sub)theme of views, barriers and strategies are presented in Appendix D in S1 File.

**Table 2:** Overview of the perceived barriers and potential strategies regarding substitution of care as expressed by dermatologists and GPs in the interviews.

	Dermatologists	GPs
Perceived barriers	(1) Lack of trust in GPs to perform skin cancer care (2) Preference of patients for a dermatologist as opposed to GP	(1) Lack of confidence in own knowledge and skills regarding skin cancer care (2) Limited communication and collaboration between GPs and dermatologists (3) Lack of trust from patients and dermatologists in GPs (4) Limited time and financial compensation
Potential strategies	(1) Expanding GP education and training in dermatology and skin cancer care (2) Need for primary care skin cancer management guideline (3) Improving and structuring the collaboration between primary and secondary care (4) Increase trust of patients in their GP by improving public relations	(1) Extending education and training for GPs in skin cancer care (2) Implementation of primary care guideline and accompanying working arrangements (3) Improving and structuring the collaboration between GPs and dermatologists (4) Efforts directed to dermatologists (5) Compensation in time and financial interventions

**General views**

Dermatologists vary in their perceived need for substitution of low risk BCC care towards primary care. Some consider substitution as a necessity for the sustainability of healthcare, mainly due to rising costs and patient volume:

“*Yes*, *it is*, *it is becoming a larger group*, *all together*, *especially the non-melanoma*, *eh the risk that your consultations are completely filled with these*, *the dermatologist cannot handle this alone*. *So as the number of patients rises*, *you need to find other solutions*.*”*

(Dermatologist interview 12)

Others feel that skin cancer care needs to be maintained in secondary care as dermatologists remain the experts regarding this type of care. Dermatologists holding positive views regarding substitution state that certain conditions (e.g., improved education and training program of GPs–see potential strategies) need to be met first:

*“Well*, *it all depends on education*, *I believe it could belong to primary care but not at this moment*, *because GPs are inadequately educated (…) especially for skin cancer*.*”*

(Dermatologist interview 2)

Moreover, some dermatologist believe that substitution to primary care is only suitable for certain parts of the care process, such as follow up care and prevention, which need to be demarcated clearly. Furthermore, it is questioned if substituting low risk BCC care should be a priority, since it is one of the many things health policy makers are trying to shift towards primary care:

*“I am not sure if the GPs are waiting for this*, *it is a political movement that everything needs to be shifted towards primary care*. *The question is if the primary care can handle all of this*.*”* (Dermatologist interview 15)

Some dermatologist express major concerns regarding this substitution potential, as dermatologist remain the experts and they doubt whether GPs can adopt this care without loss of quality. They doubt that substitution leads to less expenditures, as they believe it will lead to unnecessary excisions performed by GPs, whereas dermatologist could provide quick and accurate care:

*“If I need to do things over that have gone wrong in primary care*, *it eventually will cost more money*. *It is not my intention to kick against primary care*, *because I do respect the GPs*, *but it has to do with what you can expect from primary care*? *And that has to do with how simple*, *how straightforward do you see skin cancer*. *I think it is more difficult than a lot of people think*.*”*

(Dermatologist interview 6)

**Perceived barriers**

**Lack of trust in GPs to perform skin cancer care**

Dermatologists report a lack of trust in GPs to perform skin cancer care as a first barrier to the substitution of care process. They state to have negative experiences regarding GPs’ ability to diagnose and treat cutaneous lesions accurately. However, dermatologists also report that there is a wide variation in trust in GPs, as a result of a variation in expertise:

*“All the GPs should have sufficient knowledge*, *and before that*, *I don’t know*. *There are good GPs who can do it*, *but there are also many GPs from who you can see from the referral notes that they don’t have a clue*.”

(Dermatologist interview 16)

One of the reasons dermatologists report to be the cause of their experienced inability of GPs, is the limited and restricted education and training in skin cancer management that GPs receive. This applies to medical school, specialty GP training as well as to continuing medical education (CME):

*“There is really little attention for dermatology in the GP training*, *while 1 in 5 problems is related to the skin*. *That is just horrible*, *in 3 years of training*. *Last year I gave an additional training to GPs*, *and you notice by the questions they ask that there is a need for continuing education*, *and that they are interested in dermatology generally speaking*, *but they don’t have an anchor because they are not educated*.”

(Dermatologist interview 5)

Another reason for the experienced inability of GPs reported by dermatologists is that patient volume of low risk BCC in primary care is restricted. Dermatologists state that GPs do not see enough patients in order to become more experienced with its management:

“*It has to do with the fact that GPs need to do all sorts of things*. *I’m not afraid to say this because I was a GP before*. *GPs are just not capable*, *it doesn’t speak to them*, *because they don’t see it often enough (…)*.*”*

(Dermatologist interview 6)

**Preference of patients for a dermatologist as opposed to GP**

A second barrier reported by the dermatologists is that patients often report to prefer a dermatologist rather than a GP. Dermatologist state some patients express limited trust in GPs; when the skin lesion was initially not recognized as skin cancer by the GP, this causes the patient to lose confidence in the ability of GPs. This may complicate the substitution process:

“*It really depends on how they [patients] were initially treated*. *Because often patients were already worried about the lesion in the beginning*, *and it was downplayed by the GP*. *Then they find it hard to be referred back to the same person because they don’t trust them anymore*, *so that makes it harder*.*”*

(Dermatologist interview 3)

**Potential strategies**

**Expanding GP education and training in dermatology and skin cancer care**

One of the main strategies suggested by dermatologist to facilitate the substitution of low risk BCC care, is expanding and improving the education of GPs in both general dermatology and in skin cancer care specifically. According to dermatologists, this needs to be expanded in both in specialist GP training as well as in CME. In addition, gaining expertise through experience was reported to be important as well:

“*You need to educate them*, *to learn them*, *(…) is it difficult though*, *because it is complicated*, *also for us to make the right diagnosis*, *let alone for a GP*. *But if it is really necessary*, *then something needs to change I think*. *You need to realize that an average GP only has a few weeks of dermatology in the specialist training*, *that’s really shocking*.*”*

(Dermatologist interview 7)

Some dermatologists believe that it is essential to educate all GPs, whereas others think education should be targeted to a particular group of GPs:

“*You can think of a GP with accredited specialization for skin cancer*, *and that physician can then hold up the skills and pass on new things*. *(…) It is a select group*, *but the point of this is that he doesn’t operate solitary*, *but to serve as a contact person*.*”*

(Dermatologist interview 10)

**Need for primary care skin cancer management guideline**

A second strategy to facilitate substitution mentioned by dermatologists is the issuing of a primary care guideline on skin cancer to provide GPs guidance in skin cancer management (this was not yet available when the interviews were conducted):

“*It [the GP guideline] is their only guidance*, *so let’s hope it at least has an influence*.*”*

(Dermatologist interview 5)

Moreover, some dermatologists expressed that a national primary care guideline could provide a basis for creating local working arrangements between primary and secondary care.

**Improving and structuring the collaboration between primary and secondary care**

A third strategy to facilitate substitution proposed by dermatologists is to improve and structure the collaboration between primary and secondary care.

“*If GPs are taking over the larger part*, *then I think the communication needs to improve*. *The GP will then be more comfortable with what he does*.*”*

(Dermatologist interview 1)

Implementing a joint consultations model of care (i.e., outreach model) was also mentioned by dermatologists in this respect. This model refers to specialists providing services in a primary care setting. According to some dermatologists, in this way, GPs can become more educated and skilled, making the transition of substitution more fluent:

“*I think that a specialist outreach would work well*. *(…) If you would do that for a year*, *that after that you can easily say I send patients back*, *the GP can do this himself*.*”*

(Dermatologist interview 13)

Providing a safety net for GPs was also suggested by dermatologists. This could provide a means to give dermatologists a sense of control in assuring them that GPs are providing good quality care.

**Increase trust of patients in their GP by improving public relations**

A fourth strategy to facilitate substitution as mentioned by dermatologists is to increase patients’ trust in GPs. As mentioned earlier, patients who are managed by a dermatologist may also impede the potential for substitution as they want to remain in secondary care because of a limited trust in their GP. To tackle this barrier, it was suggested that both media and dermatologists should spread a more positive view regarding GPs’ skin cancer management:

“*Recently*, *there is a lot of attention for skin cancer in the media*, *including messages that GPs recognize it too late*, *that of course doesn’t help*. *I think that not only education is important but also some marketing or PR for the GPs like ‘hey we also join in the management of skin cancer’*. *Maybe also from the perspective of the dermatologist*, *like we do it as a team together with the GPs*.*”*

(Dermatologist interview 4)

#### Views of GPs

Four barriers and five strategies concerning substitution of BCC care were identified in the data for GPs, aside from their general views (Table 2). Additional quotations of each (sub)theme of views, barriers and strategies are presented in Appendix D in S1 File.

**General views**

The GPs are largely positive and amendable for the potential of substituting (low risk) BCC care towards primary care. Most GPs believe that this care is suitable for GPs, and that substitution is desirable considering increasing health care costs and waiting periods in secondary care. However, the GPs also state that before this can be implemented, certain conditions need to be met, such as improving education (see potential strategies):

“*I think cutaneous malignancies are suitable for primary care*, *especially concerning the BCCs as being the largest group with rising incidence*. *I think that if we want to do more with that there needs to be attention for a real project*, *including additional education for GPs*. *And also for additional funding*, *as GPs are currently insufficient equipped for the extra work that comes with it*.*”*

(GP interview 3)

A small group of GPs is rather negative about the concept of substituting BCC care, as more and more healthcare is substituted towards primary care or as they believe that patients may be better off at the dermatologist for BCC care:

“*Look*, *if it is possible and you have a lot of GPs who like it and who want to educate themselves in dermoscopy*, *and if they can become really good with the number of patients they see*, *well yes than I have nothing against it*. *But I don’t know who has the time to become skilled*. *(…) I just don’t think that we are good enough*, *we see too little*.*”*

(GP interview 5)

**Perceived barriers**

**Lack of confidence in own knowledge and skills regarding skin cancer care**

A perceived lack of confidence of GPs in their own knowledge and skills regarding skin cancer management is considered as a first barrier for substitution. Some GPs express to have limited skills to manage skin cancer with good quality, which may result in referring low-risk cases of skin cancer:

“*(…) Insecurity of the physicians [GPs] regarding their knowledge and insecurity of the patient regarding the physicians’ [GPs’] skills is blocking substitution*.*”*

(GP interview 14)

Furthermore, GPs also expressed lacking the knowledge on how to manage skin cancer care, resulting in management based on trial-and-error. The main reason stated by GPs for their limited sense of competence is inadequate education in dermatology, with a limited share in GP specialist training:

“*The skin is also a large topic for GPs*, *the question is raised many times*. *So it is actually unfortunate to do so little with it*. *It is only a small part of the GP specialist training*, *I think that is regrettable*. *So yes*, *I think something needs to happen there*.*”*

(GP interview 6)

**Limited communication and collaboration between GPs and dermatologists**

A second barrier to substitution of care reported by GPs is limited communication and collaboration between GPs and dermatologists. GPs report that agreements are lacking, expectations of dermatologists are unclear to GPs, and limited information is received from dermatologists when patients are referred back to primary care:

“*Right now it is a bit confusing yes*. *It doesn’t work that well*, *at least not with the dermatologists we are working with*. *Eh yes*, *this can definitely improve*.*”*

(GP interview 21)

**Lack of trust from patients and dermatologists in GPs**

A third barrier to substitution reported by GPs is a perceived lack of trust from patients and dermatologists in GPs. The GPs state they often experience that patients express their distrust in the GP and explicitly request a referral to a dermatologist. However, it was also mentioned that there is a patient group that expressly insists on being treated by the GP; mainly due to out-of-pocket expenses for patients that accompany referrals to secondary care, and patients preferring to be treated closer to home by the same physician.

Some GPs report to experience distrust and sometimes even disrespect from medical specialists, for example when not all GPs perform the same amount of a specific type of care, or when a referral appears to be unnecessary. In the experience of GPs, patients are often kept in secondary care longer than necessary as a result. According to the GPs it is desirable for dermatologists to have a better understanding of the position of the GP as a generalist:

“*(…) it causes distrust on both sides*, *if in secondary care it is noticed that some GPs do stuff that others don’t*, *they tend to label GPs that don’t do something as ‘bad GPs’*. *Of course this isn’t correct*, *because we have profession with such a broad range*, *so those GPs will often do something else more active*. *This makes is difficult to harmonise primary and secondary care*.*”*

(GP interview 20)

**Limited time and financial compensation**

A fourth barrier reported by GPs is the limited time and already heavy burden in primary care, especially since other dimensions of health care are also substituted. Furthermore, according to the GPs, limited financial compensation is hindering substitution, as it restricts GPs in the time investment needed for this substitution potential.

“*The financial compensation is always a thing*, *(…) if these patients are all coming to our consultation hours*, *our financial compensation is not anticipated for this*.*”*

(GP interview 9)

**Potential strategies**

**Extending education and training for GPs in skin cancer care**

Similar to dermatologists, one of the main strategies to facilitate substitution mentioned by GPs is extending education and training in dermatology. GPs believe that education should be extended both in medical school, specialist GP training and CME. A specific suggestion included education being presented by GP experts rather than a GP in training or a dermatologist, as they believe GPs think differently compared to dermatologists:

“*In specialist GP training there is relatively little dermatology education*, *while it is presented very often in the primary care practice*. *So that can be expanded*. *And I must say*, *what bothers me about the specialist GP training is that one GP trainee from the group just prepares a small presentation on one topic and only little money is spent on external expertise (…) talk about the blind leading the blind*.*”*

(GP 20)

Some GPs state that skin cancer care should not be imposed to all GPs, but should focus on a select group of motivated GPs, providing an argument for implementing accredited skin cancer specialization:

“(*…) I would be nice if you could fall back on someone who controls it*, *who is a master*, *because it is not hard to understand*, *but you need to have someone who takes control and leads the way*. *Someone who can show you like this is how you do it*. *Otherwise you have a referral of which you question if it was worth the referral*. *Well*, *at least*, *that is what I see*.*”* (GP interview 18)

An alternative suggestion reported by GPs is that GPs have to obtain certificates before they can perform skin cancer management:

“*I think you need to keep it tight*, *so if you don’t have enough knowledge*, *you shouldn’t be doing it*. *The only way you can prove this is by such certificates or diplomas*.*”*

(GP interview 21)

**Implementation of primary care guideline and accompanying working arrangements**

A second strategy to facilitate substitution as reported by the GPs is the issuing and implementation of the primary care guideline for skin cancer management. GPs report a high need, and expect this guideline to highly influence the GPs’ skin cancer management and facilitate substitution:

“*It always has a huge impact if there is a primary care guideline*. *It immediately boosts the knowledge and confidence of the GP*.*”*

(GP interview 20)

GPs mention the vital role of the Dutch College of General Practitioners (the organization which develops the guidelines) to support substitution. Furthermore, it is mentioned that sharing the guideline with secondary care may prove useful as it may provide a basis for local working arrangements between primary and secondary care.

**Improving and structuring the collaboration between GPs and dermatologists**

A third strategy proposed by GPs to facilitate substitution of skin cancer care is improving and structuring the collaboration between primary and secondary care. According to GPs this could be achieved by increasing inter-specialty communication, for instance by clear information on consultation results when transferring the patient back to primary care (including clear advice):

“*I think that communication*, *being able to find each other*, *not being afraid to ask questions*, *receiving feedback*, *not only on this area*, *but also on other domains*, *this remains of incredible importance*.*”*

(GP interview 15)

Creating an outreach model with joint consultations was also suggested by GPs, not necessarily as a goal itself, but mainly as a mean to educate GPs:

“*(…) It is about transferring knowledge*, *so if that means that with joint consultations you can lift this care up*, *than that would be great*.*”*

(GP interview 15)

**Efforts directed to dermatologists**

Several strategies directed to dermatologists were mentioned by GPs to facilitate substitution. First, dermatologists and their professional association need to support the concept of substituting low risk BCC care for it to be effectively implemented. To achieve this, it is important to increase the trust of dermatologists in GPs; dermatologists should therefore have better understanding of broad range of the GP profession:

“*In my opinion it is often patronizing*. *If you refer someone and then it appears to be something else then what you initially thought*, *you get a back like ‘you should have known better’*.*(…) They [dermatologists] need to realize we are generalists*.*”*

(GP interview 16)

Furthermore, they believe that dermatologist have a responsibility to refer low risk patients back to primary care, and explain to patients about the collaboration with GPs in skin cancer management to also increase the trust of patients:

“*I do feel that patients sometimes are kept in secondary care for too long*. *Then they don’t want to go back to primary care anymore as they think that to be seen by the dermatologist is necessary*.*”*

(GP interview 17)

**Compensation in time and financial interventions**

A fifth strategy to facilitate the substitution process as mentioned by GPs is to increase compensation in time and means, as this would result in aligned incentives. The GPs report that additional human resources are needed to manage these additional activities, especially with skin examinations being relatively more time consuming. It was therefore argued the restricted consultation time slots set by insurers need to be re-evaluated.

“*(…) Especially more time in primary health care*. *It is a growing population with these kind of things*. *(…) When this would not be managed in the hospital but with us than we certainly need extra man power*, *it is really time consuming*, *you’re easily 15–20 minutes occupied when you want to do a follow up consultation the right way*.*”*

(GP interview 3)

Furthermore, GPs mention that additional compensation is a reasonable request due to the existing differences in financial compensation between primary and secondary care. GPs report that with financial compensation they can assimilate this care, with for instance additional support staff:

“*For substitution of this care*, *the skin cancer care towards primary care*, *ehm well*, *conditions that need to be met are time and means*. *I want to see and do more*, *but then something has to oppose this*, *for me to assimilate it and fit it in*, *for me to pay for support staff*.*”*

(GP interview 14)

#### Practical solutions of selected GPs with noted willingness in skin cancer care

GPs in the focus groups were generally positive regarding substitution of low-risk skin cancer care towards primary care, but indicated that certain practical solutions are necessary to achieve this. Aside from improving GPs’ skin cancer knowledge, additional financial compensation and efforts from both GPs and dermatologists to keep patients in/refer them back to primary care, which they considered as obvious and essential strategies for substitution, GPs reported four practical solutions for implementing substitution all related to the organizational structure of care.

**Clustered consultation hours for skin cancer**

A first practical solution to be implemented in current GP practices reported by GPs is clustering patients in specific consultation hours. By organizing the consultation hours in a way that skin cancer patients are clustered (for example once a week), it would make it more easy for GPs to focus on this type of care and to manage it more efficiently. According to GPs, this could increase the trust of patients towards GPs.

“*Such clustered consultation hours*, *I do that*, *that definitely helps*. *People notice that you focus on dermatology*.*”* (Focus group 3)

**Implementation of a horizontal referral system**

A second practical solution proposed by GPs concerned implementing a horizontal referral system within primary care. Such a system implies GPs referring patients to a GP in the same practice or region with expertise on the specific topic. In the current referral system this is oriented vertically (i.e., from primary to secondary care). The GPs argued that by referring horizontally, GPs can focus on the type of care for which they have strong affinity. Moreover, in this way the particular GPs see high(er) numbers of skin cancer patients, to build up sufficient skin cancer care expertise, leading to high quality care.

“*In my practice I am the only one*, *I have a duo practice with my colleague*, *I do all the minor surgery*, *and eh*, *we think it is qualitatively good because I maintain a certain skill*, *and she does all the diabetics*.*”* (Focus group 1)

**Outreach model of care including joint consultations**

Implementing an outreach model was proposed as a third practical solution focusing on collaboration between primary and secondary care. In this model of care, as proposed by the GPs, dermatologists would provide outreach services in the primary care setting, ideally including joint consultations. According to the GPs, this is likely to result in improved quality of care (in terms of providing secondary care close to home) and in turn will improve GPs’ knowledge and expertise in skin cancer care.

“(*…) Think about an outreach model of care*, *that there is a dermatologist who you can see patients together with*, *that you do minor surgery together in your own practice so you can broaden you knowledge*, *and eh that makes it all just more fun and interesting and much more interactive with each other (…)*.*”* (Focus group 1)

**Practice size reduction**

A final practical solution proposed by GPs for substituting low risk BCC care was practice size reduction. This involves a structural decrease in patient population size per GP, resulting in GPs having more time per patient. Currently, GPs reported to experience time barriers for implementing skin cancer care. It was argued that with lesser patients to take care of, more time could be spent on providing (good quality) skin cancer care.

“*I mostly plan twenty minutes for a minor surgery*, *so when I have a lot of these then I could have seen twice as much patients on my consultation hour*, *so then I just get stuck*. *So I think that practice size reduction*, *that we get smaller practices*, *eh I think that is in this case absolutely necessary*.*”* (Focus group 1)

## Discussion

### Summary of main findings

In this qualitative study we have explored the views of the key stakeholders concerning substitution of low-risk skin cancer care from hospital to primary care and identified the perceived barriers and potential strategies to address these barriers. Dermatologists appeared to be more hesitative in their general views regarding substitution, whereas GPs were generally more positive and amendable. Stakeholders reported various barriers such as a lack of trust in GPs to perform skin cancer care, patient preferences for dermatologists, limited collaboration between GPs and dermatologists, and limited time and financial compensation for GPs. Potential strategies as suggested by the stakeholders included improving GP education, implementation of the primary care guideline, time and financial compensation and efforts to increase patients’ and dermatologists’ trust in GPs. Moreover, the GPs with noted willingness regarding substitution of skin cancer care suggested practical solutions which predominantly focused on changes in the current organizational structure of the health care system.

### Context with existing literature

One of the main barriers for substituting low risk skin cancer hospital care towards primary care is lack of trust in GPs to adequately perform skin cancer care. Similarly, GPs report a lack of confidence in their own skills to perform skin cancer care. Previous studies have indeed shown that GPs perform inferior to dermatologists in diagnosis and surgical excisions of BCCs.[28–32] This may largely be the result of the limited skin cancer education and training GPs receive during their medical school and GP specialist training; this was confirmed by GPs in our study and similar findings have been shown in other countries.[33–42] Not surprisingly, in addition to implementation of the primary care skin cancer guideline, improving GPs’ skin cancer education and training was suggested as a main strategy to facilitate substitution by all stakeholder groups (i.e., GPs, dermatologists, and GPs with noted willingness). This is consistent with the National Institute for Health and Clinical Excellence (NICE) recommendations, highlighting the pivotal importance of further training of GPs if they are to take on a larger role in skin cancer care.[43] Providing sufficient opportunities for lifelong learning is pivotal with the broad field of knowledge required in primary care and many actualizations in the medical field.[33, 36, 44]

Even with substantial expansion of GPs’ skin cancer education and training, some dermatologists questioned if substitution of low risk skin cancer care is feasible, primarily as they believe the patient volume as seen by GPs is not enough to build up sufficient expertise. Related to this, GPs in our study stated that substitution of care should not be mandatory for all GPs, but pleaded substitution to be dependent on the particular motivation and affinity of the GP with skin cancer management, which they believed to be essential for providing high-quality care. According to the GPs, this is inherently related to the broad working field of primary care physicians, and therefore they suggest efforts from dermatologists to increase their understanding in their broad profession. Consistent with this, GPs with noted willingness in skin cancer suggested practical solutions which mainly focused on facilitating qualitatively good substitution to a limited group of GPs, rather than to the total group of GPs. Suggestions involved implementing a horizontal referral system and an outreach model of care. Whereas the outreach model has showed promising results in terms of referrals, several international studies also questioned its cost-effectiveness.[1, 45–51] However, it should not be overlooked that besides the initial goal of lowering costs, outreach models involving joint consultations of GPs and dermatologists may provide a means of educating and motivating GPs, as well as improving collaboration between GPs and dermatologists in skin cancer care.[49, 52–55]

Increasing trust of patients and dermatologists in GPs to perform skin cancer care was reported by the stakeholders in our study to be fundamental for implementing effective substitution. Previous qualitative studies with skin cancer patients indeed identified a lack of trust in GPs among patients. [56, 57] Patients requesting referrals due to limited trust in their GP, in combination with the demand-satisfying attitude of GPs (and physicians in general), may counteract the substitution potential.[58–61] To tackle the barrier of lack of trust among patients it was suggested that both dermatologists and GPs, as well as media, should spread more positive views on the position of the GP in skin cancer management. Furthermore, dermatologists have a vital role in referring patients back to their GP, which may in turn increase GPs’ confidence and empower them to fulfil a larger role in skin cancer care.[62] Additionally, the proposed solutions of clustered consultation hours, horizontal referral systems and outreach models may also increase patients’ and dermatologists’ trust in GPs, as it emphasizes the particular GPs’ focus on skin cancer care.

Time and financial compensation were mentioned to be essential by GPs for successful substitution. As suggested by the GPs with a noted willingness in skin cancer, the time barrier could be addressed by implementing practice size reduction in primary care. In the current Dutch situation the consultation length of primary care physician consultations is restricted to 10 minutes per patient, which is among middle-ranking in international comparison.[63] Practice size reduction implies lesser patients to take care of per GP, thus creating more time per patient, which ultimately may lead to better outcomes (e.g., less referrals) and better quality of care. Although promising, thus far there is limited international evidence on the effectiveness of this strategy.[64–66] Furthermore, current physician payment models, such as capitation, are not aligned with this strategy, nor with the other proposed solutions such as horizontal referral systems or outreach models.[59, 67–69] Restructuring the current payment model with an increasing focus on quality rather than quantity, may be needed to facilitate substitution of care.[70]

### Strengths and limitations

Substitution of care requires complex changes in clinical practice and organization of care.[13, 71] The willingness to change of stakeholders is essential in this process of change, and strategies should be aligned with stakeholders’ different stages in the implementation process. These different subgroups require a different and adjusted approach, and different strategies may be needed at different phases of the implementation process of substitution of care.[10, 12] A strength of our study is that besides from including the views of the key stakeholders, we also included views of GPs in different phases of the process of substitution of skin cancer care. By including GPs with noted willingness in skin cancer care (i.e., early adopters), we were able to obtain more practical solutions to address existing barriers[72], in addition to the more common but essential strategies such as education, and time and financial compensation. An additional strength of this study is that it is a large national study using both focus groups and interviews as complementary sources.

A limitation of our study is that dermatologists may not have an accurate view of patients in terms of preferred healthcare providers, as they solely see the group of patients who were referred to secondary care. As also reported by GPs in our study, research has shown that—aside from patients preferring to be treated by dermatologists—there is also a group of patients that actually prefers to be treated by a GP.[32, 73] Although the barriers are actually perceived by dermatologist and thus relevant in determining their views on substitution of care, these findings indicate that it may be useful to provide dermatologists with a more accurate and complete view of patients’ preferences for healthcare providers, thereby contributing to the substitution of care process. Another limitation to be considered is that the conduction of the interviews was just prior to the issuing of the Dutch primary care guideline. However, the focus group meetings were conducted afterwards and indicated the persistence of similar issues. This confirms findings of previous studies that guidelines are often not uniformly applied in practice[74, 75] and implementation can take many years.[13, 74, 75]

## Conclusions and implications

Although our study suggests stakeholders to generally have moderately positive views regarding substitution of skin cancer care towards primary care, several substantial barriers need to be addressed before substitution of low-risk skin cancer care can be further implemented. Certain strategies such as effective skin cancer education for GPs, implementation of the primary care guideline, and time and financial compensation are regarded as essential preconditions. In addition, several proposed solutions focusing on rearranging the current organizational structure in primary care (e.g., horizontal referral system, clustered consultation hours) and between primary and secondary care (outreach models) may facilitate effective and safe substitution of low-risk skin cancer care. Since investments are required to overcome the reported barriers, further studies should explore whether these solutions are effective.

## Supporting information

S1 File Appendices A-D, including supporting information. (DOCX)

S2 File SRQR checklist including manuscript references. (DOCX)

## Abbreviations

BCC: Basal Cell Carcinoma
CME: Continued Medical Education
GP: General Practitioner
IQR: Interquartile Range
