# Supplementary material for: Substitution of low-risk skin cancer hospital care towards primary care: A qualitative study on views of general practitioners and dermatologists
Source: PLoS One. 2019 Mar 19;14(3):e0213595. doi: 10.1371/journal.pone.0213595 (PMC6424446; doi:10.1371/journal.pone.0213595)
Supplement: S1 File — (DOCX) [file pone.0213595.s001.docx]

# Appendices

## Appendix A

### SKINCATCH Trial

Basal cell carcinoma (BCC) is the most common type of malignancy with an incidence of 40,000 per year, which is rising every year. Adequate diagnosis and treatment of skin tumours is even more important as it puts such a great burden on the health care systems. The government stimulates primary care physicians to perform minor surgery on skin tumours. The SKINCATCH Trial was initiated in 2016 to investigate whether the quality of care for low risk BCCs provided by general practitioners (GPs) is not inferior to the care provided by dermatologists.

The study is a cluster randomized controlled trial, with the unit of randomization being the primary care practice. Participating GPs were randomized to either the intervention group, receiving intensive training (both theoretical and hands-on) in skin cancer management , or the care-as-usual group, receiving no training in skin cancer management. Blinding of GPs was not feasible, due to the nature of the intervention.

The primary objective is to evaluate the histological completeness of the excisions of low risk BCCs as a proxy for quality of care. Secondary objectives included the diagnostic accuracy of skin tumours, cost-effectiveness evaluation of substituting skin cancer care towards primary care, and patient reported outcomes (PROMs) on physician preference, treatment satisfaction and cosmetic outcome (t0, 3 months and 6 months after treatment).

## Appendix B

### Topic guide interviews GPs and dermatologists

**Introduction**

- Introduction
- Background and aim of study
- Aim and structure of interview
- Informed consent forms, permission audio-taping, demographic questionnaire to be filled in

**Views on substitution of care**

- Views on current distribution of skin cancer care
- Views on substitution of skin cancer care from hospital towards primary care
- Preferred roles of dermatologists and GPs in skin cancer care

**Barriers regarding substitution of care**

- Perceived (potential) barriers regarding substitution of care (e.g. education, collaboration between primary and secondary care, etc.)

**Needs for improvement/potential strategies**

- Perceived needs for improvement in skin cancer care/potential strategies to facilitate substitution

## Appendix C

### Focus group topic guide

**Introduction**

- Introduction
- Background and aim of the study
- Aim and structure of the interview
- Informed consent forms, permission audio-taping, demographic questionnaire to be filled in

**Part 1: Views on substitution of skin cancer care**

- General views on substitution of skin cancer care (e.g. suitable types of skin cancer care, suitable parts of the care process)

**Part 2: Perceived barriers to substitution of skin cancer care**

- Perceived barriers to substitution of skin cancer care (e.g. education, collaboration between primary and secondary care)

**Part 3: Potential solutions to facilitate substitution**

- Practical solutions to facilitate substitution of care

## Appendix D

| **Table 1: Illustrative quotes of sub themes for the views of dermatologists regarding substitution of low risk BCC care.** | |
| --- | --- |
| Sub themes | Illustrative quotes |
| General views | Varying views on the need for substitution  *“…I feel that in the framework of the costs of health care it is currently not right…”* (Dermatologist interview 13)  *“You need to do something because the number of skin cancer patients keep on rising, so you cannot follow-up on all these patients indefinitely.”* (Dermatologist interview 7)  *“Yes, especially for follow up it would give some room in the consultation hour but you can only let it fall from your hands when you are sure that the hands with whom the patient ends up with are enough qualified, and that is currently too little with GPs, skin therapists and oncologic nurses (…) but there are exceptions.”* (Dermatologist interview 3)  *“It is difficult because on one hand the numbers are increasingly enormously, we see it is becoming a large burden for our outpatient care, on the other hand when I see how the follow up schedule is then I don’t have a large bulk of people who keep on coming endlessly (…).* (Dermatologist interview 4)  *“I think it is desirable but I don’t know how to shape that.”* (Dermatologist interview 4)  *“I am not sure if the GPs are waiting for this, it is a political movement that everything needs to be shifted towards primary care. The question is if the primary care can handle all of this.”* (Dermatologist interview 15)  *“The question is if it is worth the trouble, or if it is better to educate a few extra dermatologists and to leave this care the way it is now.”* (Dermatologist interview 8)  Positive views only when certain conditions are met  *“I think that, when they see something suspicious they should refer easily, that is it. And they need fluid N2 at their disposal, clear guidelines, and some more education to be able to make good decisions.”* (Dermatologist interview 8)  *“The GP should have a role in examining the skin, and then refer. I think that is a point, they don’t do this enough at this moment.”* (Dermatologist interview 1)  *“In the ideal world the GP should treat solitary BCCs on a non-complicated location themselves. And recognize, so first the recognition, and then the treatment (…) a GP should have the skills to perform a conventional excision of a BCC if you ask me, especially those small BCC’s on the trunk, these definitely do not need to be referred.”* (Dermatologist interview 5)  *“Yes in theory GPs can treat part of the patients, but is depends on the interest of the GP, because we also see a lot of referrals that you think like that is very clear. But a GP is just not enough educated I think (…) they need to be educated well because otherwise the quality of care goes downwards.”* (Dermatologist interview 9)  *“…Only if the education improves, because in the way it is now it is just really bad. There is too little attention for skin cancer. (…) If they are capable and learned how to handle a dermoscope.”* (Dermatologist interview 3)  *“…Especially for low complex dermato-oncology, the GP can certainly do more. But then the quality needs to be good. Adequate education is needed.”* (Dermatologist interview 9)  *“There is something to say about follow up at the GP, but then they need to be able to make a good distinction between suspicious lesions or not. I think that the follow up and treatment of solitary BCCs could go to the GP, but under the condition that the education improves and also adherence to existing guidelines.”* (Dermatologist interview 8)  *“Most important is that GPs master the diagnosis of skin cancer because they are the ones seeing the patient for the first time, and need to make a good partition. But at this moment I don’t think part of this care needs to be shifted to the GP.”* (Dermatologist interview 14)  *“I think treatment and follow up of low risk BCC can be at the GP, but they need to be educated better. Now you see the weirdest things coming by. They often have no idea where they are doing surgery on.”* (Dermatologist interview 7)  Negative views on substitution of care  *“I think it is actually the task of the dermatologists yes (…) we haven’t done the specialist training for nothing right.”* (Dermatologist interview 1)  *“In the ideal world. The question is what is the starting point. If you have endless time and availability of dermatologist, then why would you deploy GPs for skin cancer care. It doesn’t seem obvious to me. The best care could be provided by the dermatologist. And I question if it is eventually less expensive if care shifts to primary care, as treatment by the dermatologist can be quick, accurate and appropriate, and the dermatologist is not that expensive.”* (Dermatologist interview 10)  *“I think it is best to keep it at the dermatologists, but then you need enough dermatologists. (…) GPs need to be educated better and then they could do the small BCCs on the torso. But it is better to maintain this at the specialist, because they know better what they are doing, know the risk of a second, can check the whole skin, and have a good story to tell the patient. In general it is more pleasant for the patient, and also better.”* (Dermatologist interview 18)  *“As long as the level of knowledge variates this much, the patient is better off at the dermatologist.”* (Dermatologist interview 15)  *“I think it is best to maintain skin cancer care at the dermatologist. I think that in general there is most expertise, it is generally cheaper and the easiest for the patient. We still see patients on a regular basis with non-radical excisions. You can consider small BCCs to be managed by the GP, but then they need to gain more expertise.”* (Dermatologist interview 18) |
| Perceived barriers | Lack of trust in GPs to perform skin cancer care  *“There is really little attention for dermatology in the GP training, while 1 in 5 problems is related to the skin. That is just horrible, in 3 years of training. Last year I gave an additional training to GPs, and you notice by the questions they ask that there is a need for continuing education, and that they are interested in dermatology generally speaking, but they don’t have an anchor because they are not educated.”* (Dermatologist interview 5)  *“Now you see the weirdest things coming by. They often have no idea what they are excising. (…) they do call us, because they panic because they don’t know.”* (Dermatologist interview 7)  *“For now yes. Then I am at least sure that they are not being over- or undertreated. Because like when the GP excised a superficial BCC at a fragile location, then I think ‘oh what a waste’, they you should have done this or that, that bothers me, you don’t want to desert the GP in front of the patient, but in your heart you think damn it.”* (Dermatologist interview 17)  *“Especially for the follow-up it would give some space in the consultation hour, but you can only let it out of your hands when you are sure that the patient is in good hands. If you now that the person the patient ends up with is qualified enough and that is not sufficient at the moment for GPs. There are exceptions who focused on this and enjoy doing it.”* (Dermatologist interview 3)  *“Well, to be honest, my experience with that is just bad, because then they perform an excision (…) and then we need to go around it and the scar becomes larger, wrong direction, eh I don’t think that is successful.”* (Dermatologist interview 6)  *“Well, it is striking that the diagnostic accuracy of the GP, from information I gain from referrals, is not so good. An excision of a BCC, that is something, they are often in the face, sometimes it is difficult to see the margins, often not radically excised when they are referred. I don’t think the treatment primarily is in the best hands at the GP.”* (Dermatologist interview 8)  *“(…) and I think that the GP is not well enough equipped at this moment to take over this care. So I think that the quality of care at this moment is better were it is now, then if you would sent all patients to GPs, because GPs are not well enough equipped to diagnose it good enough.”* (Dermatologist interview 14)  *“All the GPs should have sufficient knowledge, and before that, I don’t know. There are good GPs who can do it, but there are also many GPs from who you can see from the referral notes that they don’t have a clue. (…) So to force this with guidelines or something, I don’t know, it doesn’t seem like a good idea to me.”* (Dermatologist interview 16)  *“Yes it would be fantastic if you can educate GPs. If you see the weird things GPs do, really, they get down with high-risk BCCs in the face, so really large tumors, or operate BCC that are large or infiltrative without a previous biopsy, so they didn’t know, they do irradical excision, up to 2-3 times (…). I know it is the wish of the government to substitute low risk skin cancer to the GP, I like the idea, but I see the trouble coming from it every week (…). I think it is really not okay the way it is now.”* (Dermatologist interview 7)  *“…I think they just see it not often enough. They don’t get the hang of it.”* (Dermatologist interview 1)  *“A GP who sees only a few of these patients a month, how are they supposed to gain experience.”* (Dermatologist interview 2)  *“It has to do with the fact that GPs need to do all sorts of things. I’m not afraid to say this because I was a GP before. GPs are just not capable, it doesn’t speak to them, because they don’t see it often enough (…).”*  (Dermatologist interview 6)  *“It depends on the circumstances, on education, and on the frequency they get to see it. If you see something too little then you won’t be good at it, it is that simple.”* (Dermatologist interview 2)  *“The most important thing is, and that is of course the core business of dermatologists, is recognizing tumors. I think that a GP could take over part of the skin cancer treatment, however, I think the problem is in the recognition. And that makes it a tough case. We as dermatologist don’t do anything else all day, while for a GP this is more difficult because he sees it less often.”* (Dermatologist interview 4)  *“All the GPs should have sufficient knowledge, and before that, I don’t know. There are good GPs who can do it, but there are also many GPs from who you can see from the referral notes that they don’t have a clue. (…) So to force this with guidelines or something, I don’t know, it doesn’t seem like a good idea to me.”* (Dermatologist interview 16)  *“Especially for the follow-up it would give some space in the consultation hour, but you can only let it out of your hands when you are sure that the patient is in good hands. If you now that the person the patient ends up with is qualified enough and that is not sufficient at the moment for GPs. There are exceptions who focused on this and enjoy doing it.”* (Dermatologist interview 3)  Preference of patients for a dermatologist as opposed to GP  *“The difficulty is also that I notice that patients find it sometimes hard to go back to the GPs because the GP didn’t think much of the skin lesion at first. But that is completely understandable, it could easily happen to me.”*  (Dermatologist interview 4)  *“It really depends on how they were initially treated. Because often patients were already worried about the lesion in the beginning, and it was downplayed by the GP. Then they find it hard to be referred back to the same person because they don’t trust them anymore, so that makes it harder. But if this was not the case, yes then they get it.”* (Dermatologist interview 3)  *“Yes I think that many patients have more trust in the medical specialist than in their GP, that plays a big part, (…) they don’t feel they were helped, that is a difficult point, because GPS already need to do allot, and it is difficult to do it all, and the dermatologist is of course the specialist for the skin, so patients feel they are in the right place.”* (Dermatologist interview 10) |
| Potential strategies | Expanding GP education and training in dermatology and skin cancer care  *“The GPs just need to be educated better, that is definitely a point of improvement, preferable in the specialist training.”* (Dermatologist interview 10)  *“You need to educate them, to learn them, (…) is it difficult though, because it is complicated, also for us to make the right diagnosis, let alone for a GP. But if it is really necessary, than something needs to change I think. You need to realize that an average GP only has a few weeks of dermatology in the specialist training, that’s really shocking.”* (Dermatologist interview 7)  *“Training is possible but the frequency needs to go up and that is of course difficult, unless you have a really large practice where you can do a ‘skin-spots-consultation hour’.”* (Dermatologist interview 2)  *“I wonder what the point is of continuing training (…) I think the chance that you can make the GPs feel comfortable with premalignancies and follow-up through telling them with a PowerPoint presentation is really small. You really need to take care for specialist training, if needed a week of joining the consultation hour for oncologic patients, then you have the feeling you can see the difference.”* (Dermatologist interview 13)  *“There is really little attention for dermatology in the GP training, while 1 in 5 problems is related to the skin. That is just horrible, in 3 years of training. Last year I gave an additional training to GPs, and you notice by the questions they ask that there is a need for continuing education, and that they are interested in dermatology generally speaking, but they don’t have an anchor because they are not educated.”* (Dermatologist interview 5)  *“I mean it is not rocket science, so if you keep up with training than a GP would recognize 90% of the lesions. And if he doesn’t no, that he is not afraid to do a biopsy, then you can see what the result is.”* (Dermatologist interview 5)  *“Dermatoscopy can help the GPs for skin cancer (…) particularly for benign skin lesions to better recognize this. They need to be educated in this.”* (Dermatologist interview 11)  *“You can think of a GP with accredited specialization for skin cancer, and that physician can then hold up the skills and pass on new things. (…) It is a select group, but the point of this is that he doesn’t operate solitary, but to serve as a contact person. Although it would be better if it would be in the specialist training”* (Dermatologist interview 10)  Need for primary care skin cancer management guideline  *“In the new guideline it is stated to screen the total skin, before there was no guideline so profit can be made there.”* (Dermatologist interview 11)  *“It is their only guidance, so let’s hope it at least has an influence.”* (Dermatologist interview 5)  *“I think it has a major influence, but I don’t know if I am happy about it, but I think it can improve care. (…) I think more is going to be treated in primary care, and I am worried about that. Will these people get the optimal care, how is the signaling, can you diagnose it in early stages.”* (Dermatologist interview 11)  *“The problem remains just looking, it has nothing to do with guidelines, it all has to do with expertise. You can’t obtain expertise by a guideline. But we’ll see. (…) If you now from the guideline how to treat a BCC, but you cannot recognize a BCC, then it stops there.”* (Dermatologist interview 6)  “*I’d say, send the guidelines to the dermatologists so they can also read what it says*.” (Dermatologists interview 1)  Improving and structuring the collaboration between primary and secondary care  *“If GPs are taking over the larger part, then I think the communication needs to improve. The GP will then be more comfortable with what he does.”* (Dermatologist interview 1)  *“I think that a ‘one-line-and-a-half’ care would work well. (…) If you would do that for a year, that after that you can easily say I send patients back, the GP can do this himself. You can make really good working arrangements, but then you first need to invest on knowledge and content.”* (Dermatologist interview 13)  *“I don’t think that skin cancer care does not need to be managed from primary care but from secondary care. (…) For us it is also for the efficiency. If I go to a primary care practice in the afternoon and see patients there, it is totally not efficient, when I can see 35 patients in the same thing in my own practice.”* (Dermatologist interview 6)  *“I think you need to take a patient seriously, so why would you do ‘one-line-and-a-half’ care, we still need to take a look but with a lower price for the medical specialist. Then I think why, it is practically the same?”* (Dermatologist interview 17)  *“(…) I have seen too much of it, do you get why I thought like jee how hard it is for them to pick it up. So yes, if it happens then you need to make sure that dermatologist, well, keep an eye on them, and that there is some kind of control.”* (Dermatologist 1)  Increase trust of patients in their GP by improving public relations  *“Recently, there is allot of attention for skin cancer in the media, including messages that GPs recognize it too late, that of course doesn’t help. I think that not only education is important but also some marketing or PR for the GPs (…).”* (Dermatologist interview 4)  *“I think we need to educate GPs in order to make patient feel confident with the knowledge of GPs.”* (Dermatologist interview 13) |
| *Abbreviations: BCC, basal cell carcinoma; GP, general practitioner* | |

| **Table 2: Illustrative quotes of sub themes for the views of GPs regarding substitution of low risk BCC care.** | |
| --- | --- |
| Sub themes | Illustrative quotes |
| General views | Positive views regarding substitution  *“I do think GPs could have a larger role in skin cancer care (…) for me it seems logical, because you are confronted with it much, so you can easily gain experience. The skin is also a large topic for GPs, the question is raised many times. So it actually unfortunate to do so little with it. It is only a small part of the GP specialist training, I think that is regrettable. So yes I think something need to happen there, but I think a GP can handle this care very good. And of course, we are with ten thousands of us, while there are only like five hundred dermatologists. So that makes a big difference.”* (GP 6)  *“I think that when the consultation hours of the dermatologists at one point will be exploding if we continue like this. The number of patients is increasing, so I think it would definitely be something for the GP to take on.”* (GP 12)  *“I think the GPs want to, they enjoy doing it, they just think it is hard because the dermatologic education has been poor. But they do think it is part of their job (…) the quality just needs to increase and we need to take genuine action there.”* (GP 15)  *“It is a handful, not that much work. If a GP can do oncology it should be skin oncology.”* (GP 18)  *“It would definitely be suitable for GPs, provided that they are interested in the subject. The profession of GP has such a wide range, that it would be hard to do everything that extensively. But I do think that many GPs should be able to do this. Especially when it concerns actinic keratosis or BCCs, it think this belongs to primary care.”* (GP 3)  Larger role for GPs under certain conditions  *“I think they want to, they like it, they think it is hard because the education in dermatology has been bad, but they do feel it is something that is part of their job. (…) so the quality needs to go up and we need work on that.”* (GP 15)  *“Well, if you have a guideline or advice from the dermatologist, than it is fine by me.”* (GP 17)  *“I notice how a dermatosis is described, by one or the other makes a huge difference. (…) You directly notice if they are capable. It would help allot if there would be a guideline for primary care, which sums up what to be aware off.”* (GP 18)  *“Yes, I think GPs should have a larger part in skin cancer care. I think the consultation hours of the dermatologist will explode if we continue like this, the numbers of patients are increasing, so I think it would be something for the GP, if you can educate them well.”* (GP 12)  *“The bulk is actinic keratosis and BCC, so if we can do a piece in that, it seems logical to me. You do get confronted with it allot, so then you can gain experience with it. Skin is of course a large topic for GPs, the question runs by often, so it actually disappointing how little we do with it. But of course it is also only a small part of the GP training, you are not enough educated, so I think something needs to happen there. But I do think the GP can do it, we are with ten thousand GPs and the dermatologist only with five hundred. So that makes the difference.”* (GP 6)  *“What I like is that the medical specialist are easily reachable for example through tele-dermatology, we can get histopathological results quick and easy, these are the fundamental requirements to provide this care.”* (GP 3)  *“I think cutaneous malignancies are suitable for primary care, especially concerning the BCCs as being the largest group with rising incidence. I think that if we want to do more with that there needs to be attention for a real project, including additional education for GPs. And also for additional funding, as it is currently insufficient equipped for the extra work that comes with it.”* (GP 3)  *“I think that if the new guideline provides support that you can lift this care up, if education is well formed. I mean, you can just dump the guideline, but you need to make sure it is remembered. So there needs to be an implementation course where you can give explanations, instructions, and where you can practice.”* (GP 15)  *“There is a tsunami of these things coming, so I think we cannot avoid to do more. But then I do want to be more educated in this.”* (GP 17)  Doubtful about a larger role for GPs in skin cancer management  *“Look, if it is possible and you have a lot of GPs who like it and who want to educate themselves in dermatoscopy, and if they can become really good with the number of patients they see, well yes than I have nothing against it. But I don’t know who has the time to become skilled. (…) I just don’t think that we are good enough, we see too little.”*  (GP 5)  *“(…) with the rising incidence I think it would be fine to substitute, but I don’t know if everybody would agree with me. In allot of different areas there is a potential for substitution, so then the plate is becoming pretty full already.”* (GP 6)  *“I don’t favour substitution of skin cancer care because I think it is a bit boring. But I can imagine that this will come through at some point, due to health care savings and waiting lists.”* (GP 12)  *“For now our time is taken up by many others (…) the reality is that many specialties, like cardiology, neurology, all these issues are being substituted towards primary care. Inpatient hospital stays are shortened, so we run off our feet to shape complex care. So with all due respect, skin cancer care is not the most complex care were our attention needs to go to.”* (GP 14) |
| Perceived barriers | Lack of confidence in own knowledge and skills regarding skin cancer care  *“Well, I think we’re just not good enough, we see too little.”* (GP 5)  *“Yes I think more of this care can be handled in the GPs office, but not that simply. Insecurity of the physicians regarding their knowledge and insecurity of the patient regarding the physicians’ skills is blocking substitution. Especially regarding actinic keratosis and BCCs, a big profit can be achieved.”* (GP 14)  *“You need to feel comfortable with it, which differs individually. Just experience, knowing your way around, then you are not afraid to.”*  (GP 11)  Lack of trust from patients in GPs  *“(…) it causes distrust on both side, if in secondary care it is noticed that some GPs do stuff that others don’t, they tend to label GPs that don’t do something as bad GPs. Of course this isn’t correct, because we have profession with such a wide range, so those GPs will often do something else more active. This makes is difficult to harmonise primary and secondary care.”* (GP 20)  *“I do feel that patients sometimes are kept in secondary care for too long. Then they don’t want to go back to primary care anymore as they think that to be seen by the dermatologist is necessary. They will get mad when the file at the dermatologist is being closed.”* (GP 17)  Lack of trust from dermatologists in GPs  *“In my opinion it is often patronising. If you refer someone and then it appears to be something else then what you initially thought, you get a back like ‘you should have known better’.(…) They need to realise we are generalists.”* (GP 16)  Limited time and financial compensation  *“(…) with the rising incidence I think it would be fine to substitute, but I don’t know if everybody would agree with me. In allot of different areas there is a potential for substitution, so then the plate is becoming pretty full already.”* (GP 6) |
| Potential strategies | Extending education and training for GPs in skin cancer care  *“The attention for dermatology in the GP specialist training is not enough. We get 2 afternoons, in which dermoscopy is discussed briefly, and one afternoon about all the different skin diseases. This is just too little. (…) I think it is definitely possible to extend this.”* (GP 5)  *“There is a need for more emphasis and attention on dermatology in medical school.”* (GP 15)  *“Either way the education in dermatology as a whole as well as specifically dermo-oncology needs to improve, both in medicals school, specialist GP training, and continuing education.”* (GP 15)  *“Dermatology is restricted in the current specialist GP training. It highly depends on your trainer and how interested your trainer is in the subject. So there could be more attention for this in my opinion. Also in continuing education the selection is limited.”* (GP 3)  *“For dermatology as a profession with a wide range, the selection in specialist GP training is too restricted. I think it would be good if everyone would join a dermatologist for one week or something like that, that would be good for your education."* (GP 11)  *“In specialist GP training there is relatively little dermatology education, while it is presented very often in the primary care practice. So that can be enlarged. And I must say, what bothers me about the specialist GP training is that someone in the group just prepares a small presentation on one topic and only little money is spent on external expertise (…) talk about the blind leading the blind.”* (GP 20)  *“Education has priority. What I think that would work very well is joint consultation hours. I think that, besides education, that is the ultimate form of education.”* (GP 14)  *“Education should be provided by specialised GPs instead of dermatologists, because you just think differently compared to a dermatologist. A dermatologist wants a diagnosis, a GP wants to know if it is benign or malignant.”* (GP 6)  *“I think education needs to be improved and increased, and especially , I don’t know if that is achievable, but dermatoscopy would be good if that is integrated more in primary care.”* (GP 5)  *“Also you could say that GPs with accredited specialisation would be an option, just like with mental health care and lung diseases.”* (GP 15)  *“(…) I would be nice if you could fall back on someone who controls it, who is a master, because it is not hard to understand, but you need to have someone who takes control and leads the way. Someone who can show you like this is how you do it. Otherwise you have a referral of which you question if it was worth the referral. Well, at least, that is what I see.”* (GP 18)  *“If you don’t have enough knowledge than you shouldn’t do it. The only way you can prove it is by certificates, or diplomas or whatever.”* (GP 21)  *“We have like five to six thousand GPs in the Netherlands, so if you would have like 100 GPs with accredited specialisation, you get a significant improval of the quality of care.”*  (GP 18)  Implementation of primary care guideline and accompanying working arrangements  *“It is important that what is stated in the primary care guideline is shared with secondary care. That is always tense with that kind of guidelines, if they really can come to an agreement about it.”* (GP interview 3)  *“To know when you need to check someone, when you need to refer, you need a guideline for this to make good arrangements.”* (GP 12)  *“If it is stated in the guideline I would do it, because I like it to keep patient for myself and not refer them to the medical specialist.”* (GP 12)  *“I think the guideline would give us a push in the right direction. I have high expectations from it.”* (GP 7)  *“It would help a lot to have a primary care guideline, saying where to focus on. It would also be very good to have some sort of protocols for medical history and examination with a drop down menu.”* (GP 18)  *“GP care has a wide range, there is a wide range of diseases in guidelines. And what is noticed in the last 20 years is that with every primary care guideline issuing, the GP care was shaped more towards this guideline. So I expect that, when the guideline on skin cancer management is issued, it would give a certain frame for what GPs will offer. But there will still be GPs who don’t want to perform excisions. So with that it depends on what kind of GP you are. Everyone has his own specialties. That is inevitable.”* (GP 3)  *“There need to be clear instructions, and based on that clear working arrangements between the medical specialist and GP.”* (GP 21)  *“If it is descripted in the protocol, it would give some guidance and you can improve care. But only if you structure education in a good way. I mean, you can just dump the guideline, but you need to make sure that it sticks. So there needs to be an implementation trajectory in which you give explanations, instructions and practice it.”* (GP 15)  *“It always has a huge impact if there is a primary care guideline. It immediately boosts the knowledge and confidence of the GP.”* (GP 20)  Improving and structuring the collaboration between GPs and dermatologists  *“We need to have good arrangements with the dermatologists especially, and also enough time in primary care.”* (GP 3)  *“It would definitely work. And you feel heard as a GP, you can look together how to further improve things.”* (GP 16)  *“(…) Especially if it is in good collaboration with the dermatologists. That is considered as pleasant if you can consult each other easily, or send a picture.”* (GP 6)  *“I think that communication, being able to find each other, not being afraid to ask questions, receiving feedback, not only on this area, but also on other domains, this remains of incredible importance.”* (GP 15)  *“For me it is more than arrangements on paper. Personal contact, so that you know like hey that is the treatment, reliable, calling in between, that’s what I value more.”* (GP 7)  *“The opportunity to refer patients easily to the dermatologists when needed. The dermatologists are fine accessible in this area. So by the mean of tele-dermatology of by telephone, or just as an outpatient visit.”* (GP interview 14)  *“Education has priority. What I think that would work very well is joint consultation hours. I think that, besides education, that is the ultimate form of education.”* (GP 14)  Efforts directed to dermatologists  *“In my opinion it is often patronising. If you refer someone and then it appears to be something else then what you initially thought, you get a back like ‘you should have known better’.(…) They need to realise we are generalists.”* (GP 16)  *“I do feel that patients sometimes are kept in secondary care for too long. Then they don’t want to go back to primary care anymore as they think that to be seen by the dermatologist is necessary. They will get mad when the file at the dermatologist is being closed.”* (GP 17)  Compensation in time and financial interventions  *“A condition that needs to be met is space and therefore means. Sure, I want to do more, but there has to be some form of compensation, so I can assimilate it, and to afford my supporting staff.”* (GP 14)  *“There needs to be additional financial compensation, the way it is now it is not enough equipped for the extra amount of work that comes along with it.”* (GP 3)  *“There needs to be a compensation in means. From every medical specialty it is the question if GPs want to take it over, we can’t handle it. So there need to be some conditions en health insurers need to think about this too.”* (GP 14)  *“If you’ll get extra compensated, that is a motivation to do it really good, to be more active, so to speak.”* (GP 7) |
| *Abbreviations: GP, general practitioner; BCC, basal cell carcinoma* | |

| **Table 3: Illustrative quotes of sub themes for practical solutions reported by GPs selected from the SKINCATCH trial with noted willingness in skin cancer care.** |
| --- |
| Illustrative quotes |
| Clustered consultation hours for skin cancer patients  *“Such clustered consultation hours, I do that, that definitely helps. People notice that you focus on dermatology.”* (Focusgroup 2)  *“(…) If you have a moment during the day when you do it more structured, then it gets more easy of course because you’re more focussed.”* (Focusgroup 2)  *“In organizing your practice, like what you [GP in the focusgroup] are doing with a special consultation hour and structuring it and making sure you have all minor surgeries planned at a specific moment, that you make room for it and not just trying to do it in between, because then it never is a convenient time.”* (Focusgroup 2)  Implementation of a horizontal referral system within primary care  *“A: For the average GP there is a lack of knowledge in skin cancer management.*  *B: But you also have to think about whether it is necessary, because now you have all these collaboration. It is highly possible that one GP in a collaboration does it [skin cancer care] all, yes.”* (Focusgroup 1)  *“What should be implemented, in our region were experimenting with horizontal referrals, but there is a total lack of financial compensation. So when someone is sent to me by my colleague-GP in the same village, well there is just no financial compensation.”* (Focusgroup 1)  *“I think this is a good development that is coming, but it will totally put the structure upside-down. Yes it is fine by me, I really like doing it [skin cancer care] (…) There are also GPs that don’t like it, but in that case you would have the horizontal referral system, like ‘oh hey you like doing this and I don’t like it at all so you do this for me’.”* (Focusgroup 1)  Implementation of an outreach model of care  *“You should make an outreach model of care in which educated GPs are in an outreach clinic with a specialist, and you can send your patients to the GP over there that has affinity with this care. (…) I think that would be cheaper and maybe qualitatively better.”* (Focusgroup 1)  Primary care practice size reduction  *“You need more time (…) I think that in specialist care they have more than 20 minutes for minor surgery and they have a lot more supporting staff, and we need to do it all by ourselves in 20 minutes.”* (Focusgroup 1)  *“We are starting a pilot for more time for the patient. So for a normal consultation we will plan 15 minutes and for a double consultation 30 minutes. If I can do the minor surgeries in these 30 minutes, that is already better than what it is now, so I think time is really a barrier right now.”* (Focusgroup 1)  *“There is a time investment needed that you think like ‘well I need to put effort in it in a way that I need to adjust my other activities, you need to be a jack-of-all-trades. So you need to have affinity with it [skin cancer care] to give it the attention it needs.”* (Focusgroup 3) |
| *Abbreviations: GP, general practitioner; BCC, basal cell carcinoma* |
